# Role of Meprins to Protect Ileal Mucosa of Crohn's Disease Patients from Colonization by Adherent-Invasive *E. coli*

**DOI:** 10.1371/journal.pone.0021199

**Published:** 2011-06-16

**Authors:** Emilie Vazeille, Marie-Agnès Bringer, Aurélie Gardarin, Christophe Chambon, Christoph Becker-Pauly, Sylvia L. F. Pender, Christine Jakob, Stefan Müller, Daniel Lottaz, Arlette Darfeuille-Michaud

**Affiliations:** 1 Clermont Université, JE2526 Université d'Auvergne, Clermont-Ferrand, France; 2 INRA USC 2018, Clermont-Ferrand, France; 3 INRA, Plate-Forme d'Exploration du Métabolisme, Saint-Genès-Champanelle, France; 4 Institute of Zoology, Cell and Matrix Biology, Johannes Gutenberg University, Mainz, Germany; 5 Division of Infection, Inflammation, and Immunity, School of Medicine, University of Southampton, Southampton, United Kingdom; 6 Division of Gastroenterology, Department of Clinical Research, University of Bern, Bern, Switzerland; 7 Department of Rheumatology, Clinical Immunology and Allergology, Inselspital, University of Bern, Bern, Switzerland; Emory University, United States of America

## Abstract

Ileal lesions in Crohn's disease (CD) patients are colonized by pathogenic adherent-invasive *Escherichia coli* (AIEC) able to adhere to and invade intestinal epithelial cells (IEC), and to survive within macrophages. The interaction of AIEC with IEC depends on bacterial factors mainly type 1 pili, flagella, and outer membrane proteins. In humans, proteases can act as host defence mechanisms to counteract bacterial colonization. The protease meprin, composed of multimeric complexes of the two subunits alpha and beta, is abundantly expressed in IECs. Decreased levels of this protease correlate with the severity of the inflammation in patients with inflammatory bowel disease. The aim of the present study was to analyze the ability of meprin to modulate the interaction of AIEC with IECs. In patients with ileal CD we observed decreased levels of meprins, in particular that of meprin β. Dose-dependent inhibition of the abilities of AIEC strain LF82 to adhere to and invade intestinal epithelial T84 cells was observed when bacteria were pre-treated with both exogenous meprin α and meprin β. Dose-dependent proteolytic degradation of type 1 pili was observed in the presence of active meprins, but not with heat-inactivated meprins, and pretreatment of AIEC bacteria with meprins impaired their ability to bind mannosylated host receptors and led to decreased secretion of the pro-inflammatory cytokine IL-8 by infected T84 cells. Thus, decreased levels of protective meprins as observed in CD patients may contribute to increased AIEC colonization.

## Introduction

Crohn's disease (CD) and ulcerative colitis (UC) are the two major forms of idiopathic inflammatory bowel disease (IBD), with a combined prevalence of about 150–200 cases per 100,000 in Western countries. They are multifactorial diseases, occurring in individuals with genetic predisposition in whom an environmental or infectious trigger causes an abnormal immune response [1], [2]. Several lines of evidence suggest that bacteria play a role in the onset and perpetuation of IBD [3]. Intestinal bacteria are essential for the development of intestinal inflammation. In patients with CD, post-surgical exposure to luminal contents of the terminal ileum is associated with increased inflammation, and diversion of the faecal stream is associated with improvement [4]. The presence of intramucosal *Escherichia coli* or mucosa-associated *E. coli* with invasive properties in CD patients has been reported in independent studies performed in Europe and the United States [5], [6], [7], [8], [9], [10]. The phenotypic characterization of CD-associated *E. coli* showed that they are highly adherent and invasive, and accordingly they were termed adherent-invasive *E. coli* (AIEC) [11]. They form a biofilm on the surface of the ileal mucosa owing to abnormal expression of the specific host receptor CEACAM6 that recognizes the type 1 pili variant expressed by CD-associated *E. coli* bacteria [12], [13]. Flagella and outer membrane proteins (OMPs) act in concert with type 1 pili to promote AIEC bacteria adhesion to and invasion of intestinal epithelial cells and to induce intestinal inflammation [13], [14], [15], [16].

The intestinal mucosal surface is endowed with high proteolytic activity involving numerous types of endo- and exoproteases, thereby providing a broad substrate specificity. *MEP1A* has been identified as a genetic susceptibility factor for IBD [17], [18]. It encodes meprin α, an astacin-like metalloprotease synthesized as zymogen, which is activated by tryptic proteolytic processing [19], [20], [21]. Meprin α is secreted into the intestinal lumen or it is retained at the brush border membrane in association with transmembrane meprin β [22]. A variety of substrates that include extracellular matrix proteins, growth factors, and cytokines [23], [24], [25], [26], [27], [28] are cleaved by meprins whose biological function however is still poorly understood. The location and the proteolytic activity of meprins are evidence of functions at the interface of the host and the luminal environment. Meprin α knock-out mice were more susceptible to DSS-induced experimental colitis and underwent greater colon damage and inflammation than wild-type mice [17], [29]. Meprins may act as a mucosal defence mechanism that protects the intestinal epithelium against potential toxic peptides and also against enteric commensal and pathogenic bacteria by modulating the interaction between microbes and the host mucosa. The aims of the present study were to investigate meprin mRNA expression in ileal biopsies of CD patients since AIEC bacteria show a tropism for ileal colonization in CD patients and to analyse the role of meprins in the interaction of CD-associated *E. coli* and intestinal epithelial cells.

## Results

### Intestinal expression of meprins α and β

**Figure 1:**
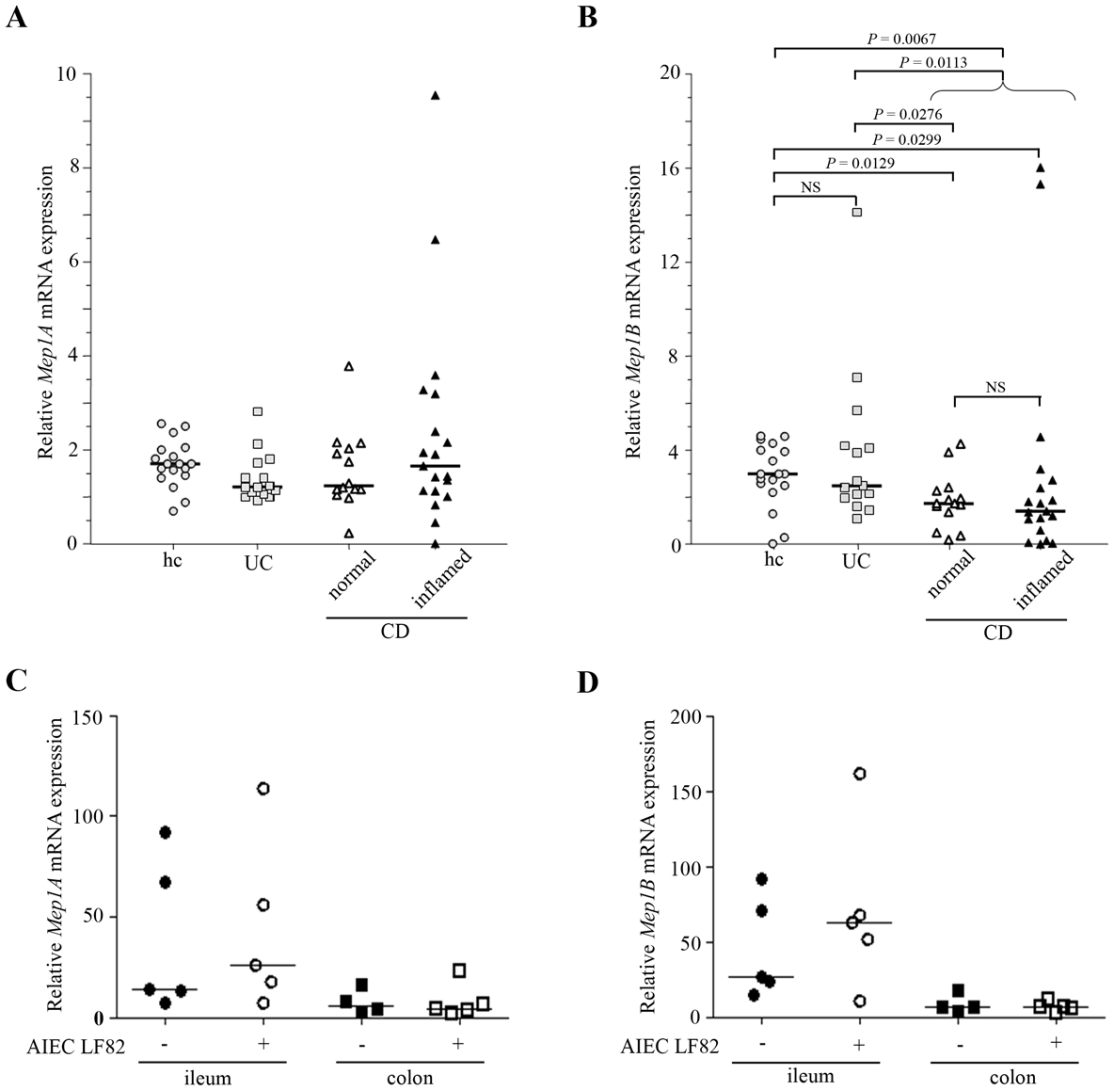
Intestinal meprin α and meprin β mRNA. A and B, Mep1A (A) and Mep1B (B) mRNA levels were determined by TaqMan quantitative real time PCR and are displayed as amounts relative to the intestinal epithelial marker villin-1. Healthy controls (hc) were compared with ulcerative colitis (UC) and Crohn's disease (CD) patients. CD patient biopsies were separated into groups with normal appearance or with macroscopic inflammation, as determined by an experienced endoscopist. Statistics were performed using GraphPad Prism 5.0 Software, and *P* values were calculated with the non-parametric Mann-Whitney test. C and D, Mep1A (C) and Mep1B (D) mRNA levels in C57Bl/6J mouse ileum and colon uninfected or infected with AIEC LF82 bacteria. The effect of AIEC LF82 infection on meprin expression was determined in mouse ileum and colon by quantitative real time PCR. Data are displayed as meprin amounts relative to the housekeeping TATA box binding protein (TBP) gene. Statistics were performed using GraphPad Prism 5.0 Software, and *P* values were calculated with the one-way ANOVA test. Dot plots show individual samples with relative mRNA (cDNA) amounts on a linear scale. Horizontal bars represent the median.

Meprin expression was analysed by quantitative RT-PCR. The level of meprin α mRNA expression was not significantly lower in the ileal biopsies of UC or CD patients than that of healthy controls (**Fig. 1A**). In contrast the level of meprin β mRNA expression was significantly reduced in ileal biopsies of CD patients, compared to that of healthy controls (**Fig. 1B, ***P* = 0.0067 Mann-Whitney U test). Interestingly, this was independent of macroscopic inflammation, as uninvolved ileal CD biopsies alone also showed significantly less meprin β mRNA expression than did controls (**Fig. 1B, ***P* = 0.0387 after Bonferroni correction for multiple testing). Ileal biopsies from UC patients showed equally high meprin β mRNA expression as healthy controls. Of note, among non-IBD controls two patients had diverticulitis and presented expression levels for meprin α (1.6 and 2.4) and β (2.7 and 3.9) close to the median, and one patient had pouchitis but the pouchitis-derived biopsy showed a rather low level for meprin α (0.8) and almost average meprin β expression (2.2).

Meprin expression was also analyzed in an adherent-invasive *E. coli* (AIEC)-induced colitis model in C57BL/6J mice. We observed that both meprin α and β were highly expressed at the mRNA level in the ileum compared to the colon (**Fig. 1C and D**). No significant modified expression of both meprins was observed following AIEC reference strain LF82 infection.

### Exposure of AIEC bacteria to meprins α and β decreased their ability to adhere to and to invade intestinal epithelial cells

**Figure 2:**
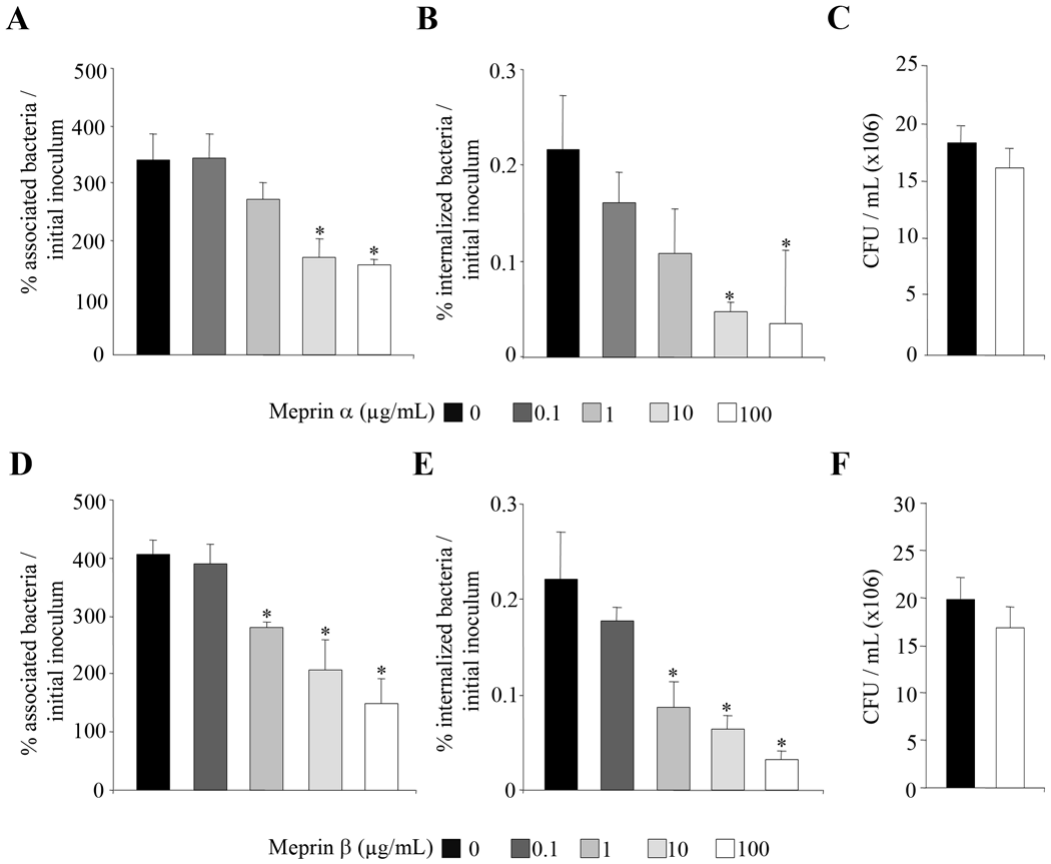
Meprins impair AIEC LF82 ability to adhere to and to invade differentiated intestinal epithelial cells. AIEC LF82 bacteria were treated before infection with increased concentrations of exogenous meprins α and β (from 0.1 µg/ml to 100 µg/ml, see Materials and Methods section). Differentiated T84 cells were infected at a MOI of 10 with untreated or meprin pretreated bacteria. A and D, the number of cell-associated bacteria was determined after a 3 h infection period. B and E, the number of internalized bacteria was determined after a 3 h infection period followed by gentamicin treatment for 1 h. Results are expressed as percentage of cell-associated (adherent + intracellular) (A and D), or intracellular (B and E) bacteria relative to initial inoculum. C and F, effect of meprin treatment on AIEC bacteria viability. Equal amounts of bacteria were exposed or not with a dose of 100 µg/ml of meprin α or β for 120 min. Thereafter, the number of viable bacteria was determined by plating on agar plate. Results are expressed as colony forming units (CFU) per ml (C and F). Data are mean ± SEM for at least three independent experiments. Student's *t*-test, * *P*<0.05.

**Figure 3:**
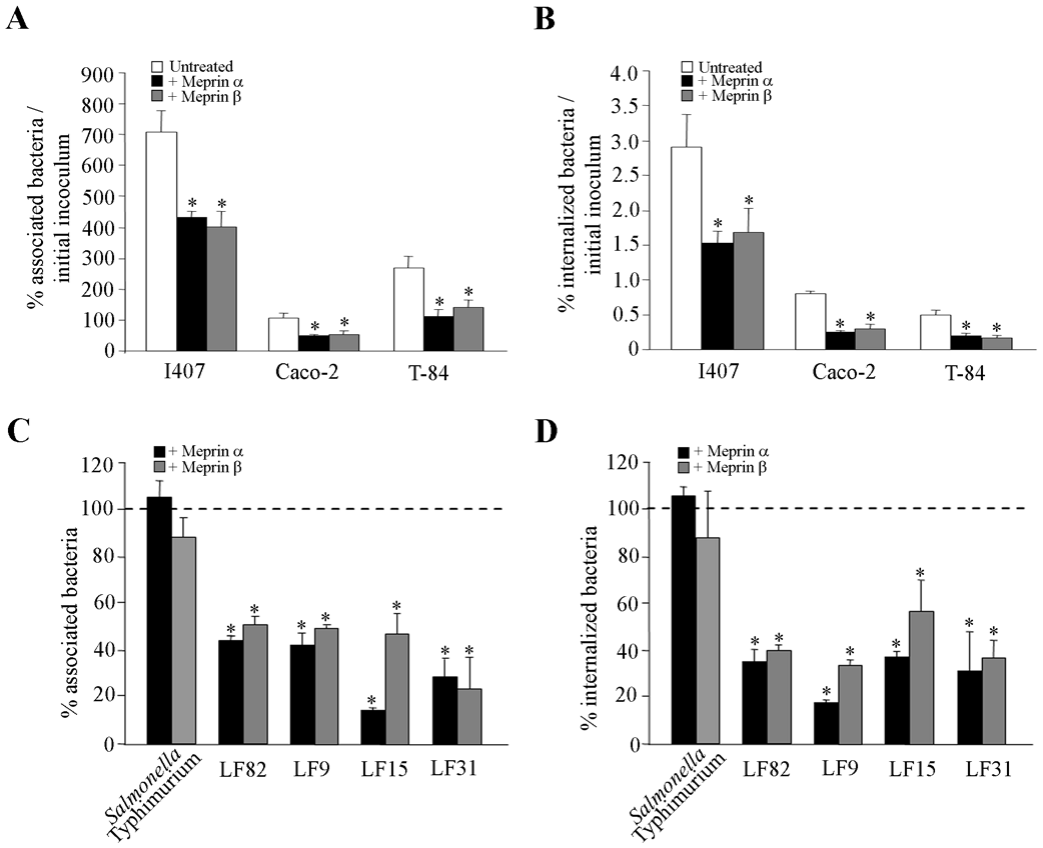
Effect of meprins on the ability of AIEC strains and *Salmonella* Typhimurium strain LT2 to adhere to and to invade intestinal epithelial cells. Bacteria were pretreated with exogenous meprin α or meprin β at 10 µg/ml. A and B, undifferentiated Intestine-407 (I407), Caco-2 and T84 cells infected with AIEC LF82 at a MOI of 10. The number of associated (A) and internalized (B) bacteria was determined. Results are expressed as the percentage of cell-associated (A) or intracellular bacteria (B) relative to initial inoculum. C and D, undifferentiated T84 cells were infected at a MOI of 10 with *Salmonella* Typhimurium and AIEC strains LF82, LF9, LF15 and LF31. The number of associated (C) and internalized (D) bacteria was determined. Results are expressed as the percentage of cell-associated (C) or intracellular bacteria (D) relative to untreated bacteria, defined as 100% (bar, C and D). Data are mean ± SEM for at least three independent experiments. Student's *t*-test, * *P*<0.05.

As endogenous meprins are synthesized in intestinal epithelial cells as inactive zymogen, which has to be activated, to investigate whether meprins can modulate the ability of AIEC bacteria to interact with intestinal epithelial cells, AIEC bacteria were pretreated with exogenous meprins α and β. The ability of the AIEC LF82 to adhere to and to invade T84 cells was assessed (**Fig. 2**). Quantitative adhesion assays showed that pretreatment of AIEC LF82 bacteria with increased concentrations (from 0.1 to 100 µg/ml) of meprins α and β significantly (P<0.05) decreased in a dose dependent manner the ability of bacteria to adhere to differentiated T84 epithelial cells, compared to untreated bacteria (**Fig. 2A and 2D**). Meprin-treated LF82 bacteria were also significantly (P<0.05) impaired in their ability to invade differentiated T84 cells, compared to untreated bacteria (**Fig. 2B and 2E**). To ensure that the decreases in adhesion and invasion levels of AIEC LF82 to cells were not due to bactericidal activity of meprins, we checked that treatment with these proteases did not affect bacterial viability (**Fig. 2C and 2F**). In addition, we observed that meprin-treated AIEC LF82 bacteria showed significantly decreased adhesion to and invasion of undifferentiated intestinal epithelial cells T84, Caco-2 and Intestine-407 (**Fig. 3A and 3B**). We also analyzed whether the effect of meprin α and β on AIEC adhesion to and invasion of intestinal epithelial cells was not limited to the AIEC strain LF82 and can be extended to other AIEC strains and other enteric bacteria such as *Salmonella enterica* serovar Typhimurium. Pretreatment of *S.* Typhimurium strain LT2 with 10 µg/ml of meprin α or β did not induce any significant decrease in either bacteria adhesion to or invasion of T84 cells (**Fig. 3C and 3D**). In contrast, for all the other AIEC strains tested (LF9, LF15 and LF31) a significant decreased ability to adhere to and invade undifferentiated T84 cells was observed when bacteria were treated with 10 µg/ml of both meprins (**Fig. 3C and D**, P<0.05). Together, these results show that meprins can modulate the interaction between AIEC bacteria and intestinal epithelial cells. We further investigated which bacterial components involved in the abilites of AIEC bacteria to adhere to and invade intestinal epithelial cells were affected by meprin treatment.

### Meprins α and β do not induce proteolytic cleavage of OMPs and flagellin

**Figure 4:**
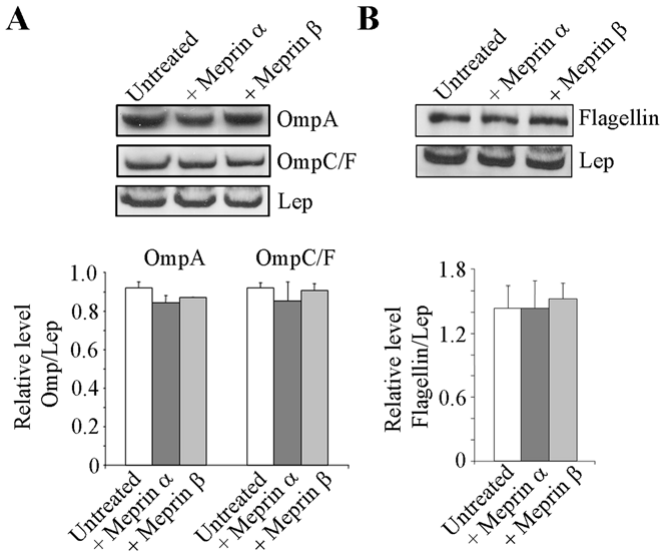
Proteolytic activity of meprins α and β on AIEC LF82 outer membrane proteins and flagellin. Total protein extracts from untreated or meprin-treated (100 µg/mL) whole bacteria were immunoblotted for OmpA and OmpC/F (A) or Flagellin (B). The inner membrane protein Lep was used as internal control. Amounts of proteins were quantified by using *Image J* software. Results are expressed as protein amount relative to Lep. Data are mean ± SEM for at least three independent experiments. Student's *t*-test, * *P*<0.05.

We have previously shown that outer membrane proteins (OMPs), by binding to the Gp96 receptor and flagella, are involved in the interaction of AIEC bacteria with intestinal epithelial cells [14], [16], [30], [31]. Meprins α and β at 100 µg/ml had no proteolytic effect on LF82 OMPs as shown by Western blot analysis using anti-OmpA, and OmpC/F antibodies and standardization against the inner membrane protein Lep (**Fig. 4A**). Similarly, treatment of LF82 flagella with meprins α or β (100 µg/ml) did not induce any proteolytic degradation of flagella as observed by Western blot analysis of flagellin (**Fig. 4B**). Together these results show that at the highest dose of meprins, the decreased abilities of AIEC bacteria to adhere to and invade intestinal epithelial cells were not due to degradation of LF82 OMPs or flagella.

### Meprins α and β induce proteolytic cleavage of AIEC LF82 type 1 pili

**Figure 5:**
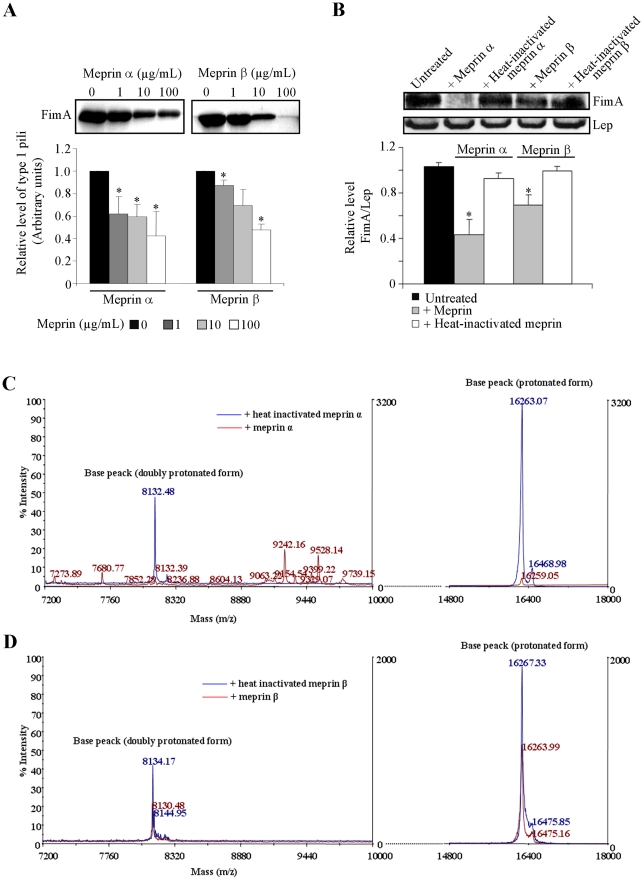
Proteolytic activity of meprins α and β on AIEC LF82 type 1 pili. A, proteolytic effect of various concentrations (from 1 µg/ml to 100 µg/ml) of meprins on purified AIEC LF82 type 1 pili. Proteins were loaded on 15% polyacrylamid gel for SDS-PAGE and stained with Coomassie brilliant blue. Results are expressed as FimA protein amount for meprin-treated type 1 pili relative to that of untreated type 1 pili. B, proteolytic effect of meprins on type 1 pili expressed at the surface of whole AIEC LF82 bacteria. AIEC bacteria were untreated or treated with active or heat-inactivated meprins at 100 µg/mL. Total proteins were immunoblotted with rabbit antiserum raised against purified type 1 pili and the inner membrane protein Lep, as an internal control. Results are expressed as FimA amount relative to Lep. Amounts of proteins were quantified by using *Image J* software. Data are mean ± SEM for at least three independent experiments. Student's *t*-test, * *P*<0.05. C and D, MALDI-TOF MS profile of purified AIEC LF82 type 1 pili treated with active or heat-inactivated meprin α or meprin β. Spectra were acquired in a mass spectrometer MALDI-TOF from LF82 type 1 pili treated with 100 µg/ml of meprin α (C) or with meprin β (D).

Experiments performed *in vitro* with cultured intestinal epithelial cells, *ex vivo* with human isolated enterocytes from CD patients, or *in vivo* using transgenic mice expressing the human CEACAM6 receptors showed that type 1 pili play a key role in the ability of AIEC bacteria to adhere to and invade intestinal epithelial cells [12], [13], [15]. We investigated whether the decrease in the abilities of AIEC bacteria to adhere to and invade epithelial intestinal cells observed after pretreatment of bacteria with meprin α or meprin β could be the result of proteolytic degradation of type 1 pili by these proteases. A dose-dependent proteolytic degradation of FimA, the major subunit of type 1 pili, was observed after treatment of AIEC LF82 purified type 1 pili with meprins α and β (**Fig. 5A**). Treatment of LF82 purified type 1 pili with 100 µg/ml of meprin α or meprin β induced a strong decrease in the FimA band observed on SDS-PAGE compared to untreated type 1 pili (89% for meprin α and 72% for meprin β). We also analyzed the proteolytic activity of meprins on type 1 pili present on the surface of whole bacteria. The amount of FimA subunit relative to that of the inner membrane protein Lep was determined by Western blot after treatment of LF82 bacteria with active or heat-inactivated meprin α or β. We observed 58% and 34% decreased amounts of FimA after treatment of the bacteria with active meprins α and β, respectively compared to those of untreated bacteria (**Fig. 5B**). In contrast, when bacteria were treated with heat-inactivated meprins the amount of FimA was unchanged. The degradation products of purified AIEC LF82 type 1 pili following meprin treatment were analyzed by mass spectrometry. Treatment of purified LF82 type 1 pili with 100 µg/ml of active meprins α and β, compared to heat inactivated meprins, modified the mass spectrometry profile of type 1 pili (**Fig. 5C and 5D**). A decrease in the intensity of the base peak of spectrum corresponding to the protonated form ([M + H]+) of major analyte detected between 16259 and 16267 m/z was observed after treatment with meprins. This was no longer observed with heat inactivated meprins. The same observation was made with the doubly protonated form ([M *+* 2H]2+) of this analyte, detected between 8130 and 8134 m/z after treatment with active meprins. Together, these results indicate that type 1 pili are a major bacterial target of meprins and that the decreased adhesion and invasion of AIEC bacteria to intestinal epithelial cells after pretreatment of bacteria with exogenous meprins could be a direct consequence of the proteolytic activity of these proteases against type 1 pili.

### Meprins affect mannose residue recognition mediated by AIEC LF82 type 1 pili and the ability of AIEC bacteria to induce IL-8 secretion

**Figure 6:**
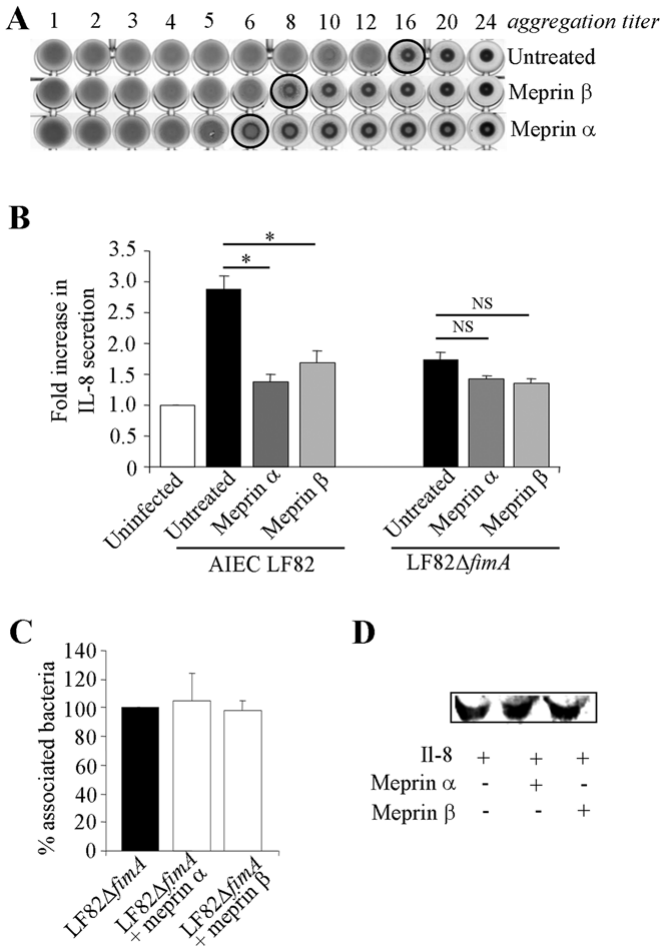
Meprin treatment affect mannose residue recognition by AIEC and AIEC-induced IL-8 secretion by T84 cells. A, ability of type 1 pili to bind D-mannose residues as determined by a yeast aggregation test. AIEC LF82 bacteria were treated with 100 µg/ml of meprin α or β at 37°C for 120 min. A fixed amount of inactivated yeast cells (*Saccharomyces cerevisiae*) suspension and decreasing concentrations of treated and untreated bacteria were mixed, and the loss of the ability to form homogenous aggregation was used as the read-out for impaired type 1 pili-yeast interaction. B, Amount of IL-8 secreted by uninfected or AIEC LF82- or type 1 pili negative mutant LF82-Δ*fimA*-infected T84 cells, at 24 h post-infection. AIEC LF82 and LF82-Δ*fimA* bacteria were treated with 100 µg/ml of meprins. Il-8 secretion was determined by ELISA. Data are expressed as fold increase in the amount of secreted IL-8 ± SEM by T84 cells infected with untreated or treated bacteria relative to non infected cells. Student's *t*-test, * *P*<0.05 for comparison between IL-8 secretion induced by untreated versus meprin-treated AIEC LF82 or LF82-Δ*fimA* bacteria. C, LF82-Δ*fimA* bacteria were pretreated with exogenous meprin α or meprin β at 100 µg/ml and undifferentiated T84 cells were infected at a MOI of 10. The number of associated bacteria was determined. Results are expressed as the percentage of cell-associated bacteria pretreated with exogenous meprins relative to untreated bacteria, defined as 100%.D, effect of meprins on recombinant human IL-8. Recombinant human IL-8 (110 ng/ml) was treated with meprin α or β (100 µg/ml), electroblotted and detected with mouse anti-human IL-8.

Adhesion of AIEC bacteria to cells that involved type 1 pili is mediated by recognition between the FimH adhesin, located at the tip of the pilus, and mannose residues on cellular receptors [32]. The ability of LF82 type 1 pili to bind D-mannose residues was tested using a yeast aggregation assay. Pretreatment of bacteria with meprin α and meprin β strongly reduced their ability to induce yeast aggregation (**Fig. 6A**). The impaired recognition of mannosylated receptor by AIEC bacteria could result from meprin-mediated proteolysis of type 1 pili.

Pathogenic *E. coli* interaction with host cells generally induce enhanced epithelial cell IL-8 secretion [33], [34]. We quantified by ELISA the levels of IL-8 secreted by T84 cells infected with AIEC LF82 bacteria pretreated or not with 100 µg/ml of meprin α or β (**Fig. 6B**). The amount of IL-8 secreted by T84 cells infected by meprin α- or meprin β-treated bacteria was significantly lower than that of cells infected with non-treated bacteria (24% and 28%, respectively, P<0.05) and was similar to the amount observed with T84 cells infected by a non-piliated LF82 mutant (LF82Δ*fimA*). To analyse whether bacterial components inducing IL-8 secretion other than type 1 pili susceptible to meprin degradation could exist, we performed assays with LF82Δ*fimA* bacteria treated with meprins (**Fig. 6B**). IL-8 secretion by T84 cells remained similar following infection with LF82Δ*fimA* bacteria treated or not with meprins. In addition, we did not also observe any modified adhesion to T84 cells of LF82Δ*fimA* mutant treated or not with meprins (**Fig. 6C**). Decreased IL-8 amounts as measured by ELISA were not due to proteolytic degradation of IL-8 by meprins (**Fig. 6D**). Thus, these results demonstrate that meprins affect AIEC interaction with IEC, since degradation of type 1 pili by meprins impaired recognition of D-mannose residues by LF82 bacteria and subsequently decreased the ability of AIEC LF82 to adhere to and invade IEC, resulting in decreased IL-8 secretion.

## Discussion

According to the currently accepted hypothesis, both UC and CD result from a dysregulated response of the intestinal immune system to antigens of microbial origin or pathogenic bacteria in genetically predisposed individuals. *MEP1A* has been identified as a genetic susceptibility factor for IBD [17], [18]. It encodes meprin α, a metalloprotease highly expressed in the intestine. Meprin α is secreted into the intestinal lumen or accumulates at the apical brush border membrane of polarized epithelial cells retained by meprin β. Thus any decrease in meprin α or β expression can lead to similar defects in the host. In the present study, we analyzed meprins α and β mRNA expression in ileal biopsies of CD patients since AIEC are more frequently found in intestinal tissue samples of CD patients, than in those of UC patients or healthy controls and because AIEC bacteria show a tropism for ileal colonization. Our real time PCR analysis with patient biopsies revealed that, irrespective of macroscopic inflammation, the ileal mucosa of CD patients had significantly weaker expression of meprin β than that of non-IBD control biopsies. As meprin β is required to retain meprin α, this may aggravate the deficit in meprin α/β secretion/retention on the luminal side of the epithelial cell membrane. In addition, we analyzed whether AIEC infection could modulate meprin expression and observed no modified expression of either meprin following AIEC LF82 mouse infection, indicating that the exacerbation of colitis by infection did not interfere with meprin expression.

We speculated that defects in meprin expression on the surface of the ileum could affect microbe-host interactions since proteases could create a proteolytic environment which either kills bacteria or degrades their virulence factors. This was well exemplified for neutrophil elastase, which induces considerable degradation of *E. coli* OmpA [35] or *Pseudomonas aeruginosa* flagellin [36], and for lactoferrin, which cleaves IgA1 protease protein and Hap adhesin of *Haemophilus influenzae*, [37]. We show that pretreatment of AIEC bacteria with exogenous meprins α and β impaired the ability of bacteria to adhere to and invade various differentiated or non differentiated intestinal epithelial cells, indicating that any decrease in meprin expression in the gut could result in increased AIEC colonization. This indicated that meprins can participate in modifications of the bacterial population associated with the gut mucosa. Interestingly, no decreased adhesion or invasion was observed with the enteroinvasive pathogen *S.* Typhimurium strain LT2 treated with meprins, probably because these bacteria use a type three secretion system to invade epithelial cells

We previously reported that AIEC colonization of the intestinal mucosa is dependent on binding of type 1 pili to the glycosylated CEACAM6 receptor, which is abnormally expressed by ileal epithelial cells in CD patients [12]. We also previously reported that flagella and outer membrane proteins (OMP) via outer membrane vesicles are involved in the ability of AIEC bacteria to adhere to and to invade cultured IEC *in vitro* [14], [30], [31]. We investigated whether meprins target these virulence factors and observed a proteolytic degradation of AIEC LF82 type 1 pili by meprins α and β, but not of flagella and OMPs. This effect was found with active meprins but no longer observed when bacteria were treated with heat-inactivated meprins. The impaired ability of meprin-treated bacteria to adhere to and invade various intestinal epithelial cells could result from proteolytic degradation of type 1 pili. Previous molecular dissections of virulence factor expression in the AIEC reference strain LF82 suggested that these bacteria are highly piliated under physiological conditions in the gastrointestinal tract [16]. Any proteolytic degradation of type 1 pili could modify the behaviour of the AIEC bacteria within the host, and any decrease in protease expression could therefore increase AIEC colonization of the ileal mucosa.

AIEC infection of IECs induces the secretion of high amounts of the pro-inflammatory cytokine IL-8, a potent chemoattractant for neutrophils [7]. We observed that when AIEC LF82 bacteria were treated by meprin α or β, IL-8 secretion by infected IECs was significantly decreased. This effect was probably due to the decrease in the ability of bacteria to adhere to and invade intestinal epithelial cells as a consequence of proteolytic degradation of type 1 pili. These results are in accordance with our previous study showing that the inflammation induced by AIEC infection of mice expressing the human CEACAM6 was not observed when the AIEC LF82 type 1 pili–negative mutant was used [13]. It can be speculated that down-regulation of meprin expression observed in CD patients leading to impaired meprin secretion/retention at the epithelial brush border might favor not only colonization of the intestinal mucosa by AIEC bacteria but also AIEC-induced inflammation of the gut.

## Materials and Methods

### Patients and controls

CD: n = 27, mean age 41 years (25-88), 12 male and 15 female patients. UC: n = 6, mean age 45 years (21–65), 2 male and 4 female patients. Controls: n = 18, mean age 55 years (38–74), 9 male and 9 female non-IBD individuals/patients. Biopsies were collected during routine (patients) or screening (controls) endoscopic examination at the Gastroenterology unit, University Hospital Bern, Bern, Switzerland. Biopsies were chosen solely upon their location and macroscopic appearance, as judged by the experienced endoscopist. CDAI and Mayo scores, although determined routinely, were not used to stratify the cohort of patients used in the present study. Informed written consent to use biopsies for the present study was obtained from all patients, and all procedures were approved by the ethical committee of the local authorities of the Canton of Bern.

### Mouse model and infection

Nine-week-old C57BL/6J (B6) mice (≈19.5 g) were bred and reared at the central animal facility of the medical faculty of the University of Bern, Switzerland. They received DSS (MW = 36,000–50,000, MP Biomedicals, Illkirch, France) in drinking water for 7 days with 3.5% (w/v) DSS for the first 3 days and 2.5% (w/v) DSS for the last 4 days. The mice were orally challenged by 5 mg of streptomycin (Sigma) at day 3 and by 10^8^ AIEC LF82 bacteria at day 4. They were sacrified at day 7 and the ileum and colon were collected. Animal experiments were approved by the local authorities of the canton of Bern and were also in accordance with the Committee for Research and Ethical Issues of the IASP.

### Real-time mRNA quantification

Human ileal biopsies and mouse ileum and colon were placed in RNAlater (Qiagen, Hombrechtikon, Switzerland). Samples were kept for 24 h at 4°C and then at −20°C until RNA extraction. RNAlater-preserved biopsies were homogenized in RLT buffer (Qiagen) in a TissueLyzer™ (Qiagen) and RNA were isolated using the mirVana™ (mi)RNA Isolation Kit (Ambion, Applied Biosystems, Rotkreuz, Switzerland). RNA quality was checked with Agilent Bioanalyzer Nano Chips (Agilent Technologies, Basel, Switzerland). One µg of RNA was reverse transcribed using Promega's ImProm-II Reverse Transcription System (Promega, Mannheim, Germany) with random primers. Meprins α and β mRNA were quantified from human samples using intron-spanning TaqMan gene expression assays (Applied Biosystems) and from mouse samples using Light Cycler 1.5 (Roche Diagnostics) and SYBR green Taq ReadyMix (Sigma, St. Louis, MO) with specific human and mouse oligonucleotides. Each sample was run in duplicate. Results were normalized to the human epithelial marker villin gene or mouse TATA box binding protein (TBP) housekeeping gene.

### Bacterial strains

The four AIEC strains (AIEC reference strain LF82 and AIEC strains LF9, LF15 and LF31) were isolated from patients with Crohn's disease [6], [11]. The LF82-Δ*fimA* isogenic mutant does not synthesize type 1 pili [38]. *Salmonella* Typhimurium strain LT2 was purchased from ATCC (ATCC 700720). Bacteria were grown routinely in Luria Bertani (LB) broth or on LB agar plates overnight at 37°C.

### Cell culture

The intestinal epithelial cell lines T84 (ATCC, CCL-248), Intestine-407 (ATCC, CCL-6) and Caco-2 (ATCC, HTB-37) were maintained in an atmosphere containing 5% CO_2_ at 37°C in the culture medium recommended by ATCC. For infection assays, undifferentiated T84, Intestine-407, and Caco-2 cells were seeded in 24 well plates at a concentration of 4.10^5^ per cm^2^. To obtain differentiated T84 cells, cells were seeded onto Transwell filters at 8.10^5^ cells/filter (5 µm pore size, 4.6 cm^2^; Costar, Corning Inc.) and were grown for 21 days in an atmosphere containing 5% CO2 at 37°C.

### Adhesion and Invasion Assays

Before infection, bacteria were pretreated for 120 min with 0.1 µg/ml to 100 µg/ml of exogenous meprin α or β in PBS (See paragraph below). Meprins were then inactivated, and pretreated bacteria were washed and used for infection. Cells were infected at a multiplicity of infection (MOI) of 10 bacteria per cell. Adhesion and invasion assays were performed as previously described [6]. For adhesion assays, after 3 h of incubation period at 37°C, monolayers were washed five times in phosphate buffer saline (PBS). To determine the numbers of intracellular bacteria (Invasion assay), cell culture medium containing 100 µg of gentamicin per ml was added for 1 h to kill extracellular bacteria. The epithelial cells were then lysed with 1% Triton X-100 (Sigma) in deionized water. Samples were diluted and plated onto LB agar plates to determine the number of colony-forming units (CFU).

### Bacterial viability assay

Bacteria were pretreated with 100 µg/ml meprin α or meprin β (expression and purification described previously [39], [40]) at 37°C for 120 min. Meprins were then inactivated and pretreated bacteria were compared to untreated bacteria. Numbers of CFU were determined from samples diluted and plated onto LB agar plates.

### Extraction of type 1 pili and total proteins

Type 1 pili were extracted as previously described [15], [16]. Purified type 1 pili were subjected to HCl hydrolysis before SDS-PAGE analysis because fimbriae are resistant to SDS disaggregation. After an overnight incubation at 37°C in LB broth, bacteria were centrifuged and resuspended in SDS-PAGE loading buffer (2% SDS, 50 mM Tris-HCl pH 6.8, 12.5% glycerol, 400 mM β-mercaptoethanol and 0.01% Bromophenol Blue).

### Treatment of whole bacteria, purified bacterial surface components and recombinant human IL-8 with meprins

Whole bacteria were pretreated for 120 min with exogenous meprin α or β (from 0.1 µg/ml to 100 µg/ml) in PBS. PBS supplemented with 4 mM EDTA pH 7.4, was then added (20∶1) in order to inactivate meprin activity. The same protocol was used for bacteria in the absence of meprin or in presence of 100 µg/ml of meprin α and β inactivated at 100°C for 15 min. Bacteria were then washed twice in PBS by centrifugation and resuspended in PBS for infection or in SDS-PAGE loading buffer for Western-blotting.

Purified type 1 pili were treated with increasing concentrations (1 to 100 µg/ml) of meprin α and β, at 37°C for 120 min and subjected to SDS-PAGE and mass spectrum analysis. In addition, purified type 1 were also treated with 100 µg/ml of meprin α and β inactivated at 100°C for 15 min.

Recombinant human IL-8 (110 ng/ml, R&D systems) was treated with 100 µg/ml of meprin α or β at 37°C for 120 min and was resuspended in SDS-PAGE loading buffer for Western-blotting.

### Western blotting

Purified bacterial surface components or total proteins were subjected to SDS-PAGE on 12-17% gels. Protein concentrations were determined by Bradford assay and the gels were stained for protein with Coomassie brilliant blue. Western immunoblotting was performed according to the procedure of Towbin [41]. Proteins were electroblotted onto nitrocellulose membranes (Amersham International), and the membranes were immunoblotted for type 1 pili (rabbit antiserum raised against purified type 1 pilus preparations, diluted 1∶1,000), Lep (rabbit anti-Lep, diluted 1∶1,000), OmpA (rabbit anti-OmpA, diluted 1∶1,00), OmpC/F (rabbit anti-OmpC/F, diluted 1∶1,000), flagellin (rabbit anti-H1, diluted 1∶500) and IL-8 (mouse anti-human; diluted 1/250; R & D Systems). Immunoreactants were detected using horseradish peroxidase-conjugated anti-rabbit or anti-mouse immunoglobulin G antibody, ECL reagents (Amersham Biosciences) and autoradiography. *Image J* software was use to estimate protein quantity.

### Yeast cell aggregation assay

Bacteria were washed and resuspended to an optical density of 0.1 at 620 nm in PBS and treated or not with 100 µg/ml of meprin α and meprin β at 37°C for 120 min. Equal volumes of fixed commercial baker's yeast cell (*Saccharomyces cerevisiae*) suspension (10 mg dry weight/ml) in PBS and decreasing concentrations of *E. coli* suspension were used, and aggregation was monitored visually.

### Mass spectrum analysis

Mass spectra were acquired by a mass spectrometer-DE PRO (Applied Biosystems, Cortaboeuf) in positive ionization, linear MODE. Briefly, 1 µl of type 1 pili was mixed on the MALDI plate with 1 µL of Sinapinic acid matrix, using the standard dried-drop method. Spectra were then generated in the mass-to-charge ratio (m/z) range of 4,000 m/z to 20,000 m/z. Each spectrum was calibrated by calibrant protein mixture (C110, LaserBiolabs, Antibes, France).

### Enzyme-linked immunosorbent assay (ELISA)

The amount of Il-8 released in the culture supernatant was determined by ELISA (R&D systems). Cytokine concentrations were assessed according to the manufacturer's instructions.

### Statistical Analysis

Data generated from adhesion and invasion assays or ELISA were analysed by Student's *t*-test. All experiments were performed at least three times. A *P*-value ≤0.05 was considered statistically significant. Data are expressed as the mean ± SEM. Statistical analysis of data generated from RT-PCR with human biopsies and with mouse colon and ileum was performed using GraphPad Prism 5.0 Software. For data generated from RT-PCR with human biopsies, overall differences between groups were estimated with the Kruskall Wallis test and *P* values were calculated with planned posteriori tests using the non-parametric Mann-Whitney U test. P-values as indicated in figure 1B were further corrected for multiple testing by Bonferroni correction for final statements in results and discussion. For data generated from RT-PCR with mouse ileum and colon, overall differences between groups were estimated with one-way ANOVA.
